# Can inflammatory markers predict response to methotrexate in JIA? Results from the CHARM study

**DOI:** 10.1186/1546-0096-9-S1-O10

**Published:** 2011-09-14

**Authors:** H Moncrieffe, D Holzinger, S Ursu, F Patrick, L Kassoumeri, J Roth, LR Wedderburn

**Affiliations:** 1 Rheumatology Unit, UCL Institute of Child Health, London, UK; 2 Immunology Institute, University of Münster, Münster, Germany

## Background

Juvenile idiopathic arthritis (JIA) is the most common childhood rheumatological disease with an incidence of 1 in 1000. Methotrexate is the primary DMARD given to patients with JIA but approximately 30% of children fail to respond. The SPARKS CHARM study enrolls patients with JIA about to start MTX with the aim to discover predictors of response to MTX, to distinguish patients who will respond well to MTX from non-responders.

## Aim

We investigated whether serum levels of inflammatory proteins such as MRP8/14 could predict JIA responder status to MTX.

## Methods

Blood samples and clinical data including core outcome variables, were collected pre treatment and at 6 months of therapy. Serum concentrations of MRP8/14 were determined by an in-house sandwich ELISA as described (Foell D, et. al. 2004). The analysing laboratory was blinded for clinical data. The study had full ethical approval and consent. Response was defined at ACR30, 50,70 levels.

## Results

109 patients were included in this study. Patients had typical frequencies of patient subtypes and MTX responder status. Systemic patients have known high levels of MRP8/14, and this was also demonstrated in our cohort, so were excluded from this analysis. There were no significant differences between age of onset between ACR70 and NR patients. MRP8/14 levels were higher in pre-MTX samples of patients who would go onto respond to MTX as ACR70 compared to patients who would not respond. Analysis of ‘intermediate phenotype’ patients who would have ACR30 or ACR50 response, revealed that ACR30 had low MRP8/14 levels comparable to NR and ACR50 levels were similar to ACR70. The difference between NR/ACR30 and ACR50/ACR70 in pre-MTX samples was highly significant (1495ng/ml vs 2705ng/ml, p=0.008).

## Conclusion

These data suggest that MRP8/14 levels are increased in JIA patients who will go on to respond well to MTX therapy. MRP8/14 is stable without cold storage which will facilitate delivery of serum samples to specialised laboratories for testing. We conclude that used in combination with routine clinical data, e.g. number of active joints, MRP8/14 levels could have a useful role in assisting in predicting which JIA patients are likely to respond to MTX.

This study was supported by grants from SPARKS UK, The Big Lottery Fund, Arthritis Research UK and Great Ormond Street Hospital Children’s Charity. We thank patients, parents and staff at Great Ormond Street Hospital NHS Trust, London.

